# Clinical Outcomes of Traction-Free Only Peripheral Hip Arthroscopy for Cam-Type Femoroacetabular Impingement

**DOI:** 10.3390/jcm15187170

**Published:** 2026-09-15

**Authors:** Muzaffer Agir, Seyedarad Mosalamiaghili, Alireza Keshtkar, Faisal Mohd Noor, Serkan Surucu, Ibrahim Tuncay

**Affiliations:** International Joint Center, Acıbadem University, Istanbul 34638, Turkey; muzafferagir@gmail.com (M.A.); aradmosalami@gmail.com (S.M.); alireza.keshtkar.med@gmail.com (A.K.); faisalmohdnoor99@gmail.com (F.M.N.); ituncay@gmail.com (I.T.)

**Keywords:** femoroacetabular impingement, hip arthroscopy, only peripheral hip arthroscopy, traction-free arthroscopy, cam morphology, labral repair, patient-reported outcomes

## Abstract

**Background/Objectives:** Femoroacetabular impingement (FAI) is a common cause of hip pain and functional limitation in young and active patients. Conventional hip arthroscopy commonly requires traction and central-compartment access, which may contribute to traction-related neuropraxia and access-related chondrolabral injury. This study evaluated clinical outcomes after traction-free only peripheral hip arthroscopy (OPHA) for cam-type FAI with associated labral pathology. **Methods:** This retrospective cohort study included consecutive patients who underwent OPHA between November 2016 and May 2022 and had complete paired preoperative and postoperative outcome data. Outcomes included the visual analog scale (VAS) pain score, Hip Outcome Score activities of daily living subscale (HOS-ADL), Hip Outcome Score sport subscale (HOS-Sport), International Hip Outcome Tool-12 (iHOT-12), and Tegner activity score. Labral status was categorized as intact, repaired, or debrided. Paired outcomes were analyzed with the Wilcoxon signed-rank test. **Results:** Sixty-six patients were included (mean age, 38.9 ± 10.8 years; 34 female). The mean postoperative follow-up was 5.7 years (range, 3.7–9.2 years). Labral status was intact in 32 patients, repaired in 23, and debrided in 11. Secondary intervention occurred in eight patients. In the overall cohort, VAS improved from 7.0 ± 0.6 to 3.2 ± 2.5 (*p* < 0.001), HOS-ADL from 59.1 ± 1.9 to 79.2 ± 17.5 (*p* < 0.001), HOS-Sport from 45.1 ± 2.1 to 67.6 ± 21.3 (*p* < 0.001), and iHOT-12 from 48.3 ± 1.8 to 72.2 ± 19.4 (*p* < 0.001). The Tegner activity score decreased from 5.2 ± 0.7 to 4.3 ± 1.4 (*p* < 0.001). **Conclusions:** Traction-free OPHA was associated with improvements in pain and hip-specific functional outcomes in selected patients with cam-type FAI and allowed individualized labral management without routine central-compartment distraction. The retrospective design and retrospective reconstruction of baseline PROMs in a subset of patients should be considered when interpreting the magnitude of the observed improvement.

## 1. Introduction

Femoroacetabular impingement (FAI) syndrome is a well-established cause of hip pain and functional limitation in young, active patients, with cam-type morphology representing the most common deformity pattern [1,2]. Arthroscopic management has become a standard surgical approach, and systematic reviews have demonstrated significant improvements in patient-reported outcomes (PROs), including pain, function, and sport-specific measures, with a substantial proportion of patients returning to sport at the same or higher level [3,4].

Conventional hip arthroscopy, however, usually relies on traction to access the central compartment. Traction and perineal-post compression are associated with recognized traction-related morbidity, including postoperative neuropraxia affecting the pudendal and lateral femoral cutaneous nerves, and iatrogenic chondral or labral injury during central-compartment access has also been described [5,6,7,8]. Techniques that minimize or eliminate traction have therefore attracted increasing interest, with peripheral-first or limited-traction approaches reporting favorable clinical outcomes and reduced iatrogenic damage [9].

Only peripheral hip arthroscopy (OPHA) is a traction-free technique confined to the peripheral compartment. It is intended to permit cam decompression and selected labral management without routine central-compartment distraction, while also allowing dynamic intraoperative assessment of impingement correction [10]. Building on our previous experience with the OPHA technique, the present study evaluates a larger cohort with a more detailed patient-reported outcome assessment. Specifically, we aimed to evaluate pain and hip-specific functional outcomes after OPHA, describe outcomes according to labral treatment, and characterize the frequency and outcomes of subsequent interventions.

## 2. Materials and Methods

### 2.1. Study Design and Setting

This retrospective cohort study evaluated the clinical and functional outcomes of patients who underwent traction-free OPHA for symptomatic cam-type FAI. Consecutive patients treated between November 2016 and May 2022 were identified from an institutional hip arthroscopy database. The study assessed postoperative pain relief, functional recovery, activity level, and the frequency of secondary postoperative intervention after the index arthroscopic procedure.

All procedures were performed using a traction-free peripheral-compartment technique on a standard operating table. This strategy was selected to avoid mechanical distraction of the hip joint while allowing arthroscopic treatment of FAI-related pathology, labral lesions, and associated peripheral-compartment abnormalities. The study was conducted in accordance with the Declaration of Helsinki and was approved by the Acıbadem Mehmet Ali Aydınlar University Medical Research Ethics Committee in 2026 (IRB code: 2026-10/441) for the retrospective analysis and scientific use of clinical data from patients who had undergone surgery between 2016 and 2022. Patient follow-up and clinical outcome assessments were performed as part of routine clinical care and were not initiated specifically for the purposes of the present retrospective study.

### 2.2. Patient Selection

Patients were eligible for inclusion if they underwent primary hip arthroscopy using the traction-free only peripheral anterior-approach technique for symptomatic cam-type FAI and had complete paired preoperative and postoperative clinical outcome data. Eligible patients had hip pain refractory to nonoperative treatment and were considered appropriate candidates for arthroscopic management based on clinical assessment and imaging findings consistent with FAI. Nonoperative management primarily consisted of activity modification and nonsteroidal anti-inflammatory and/or analgesic medication [11]. Formal physical therapy was not routinely required as part of the treatment algorithm, and no predefined minimum duration of nonoperative treatment was used. In patients in whom the clinical presentation and radiographic findings were not clearly concordant, an intra-articular diagnostic injection was routinely performed. Temporary symptomatic improvement following the injection, followed by recurrence of symptoms, was considered supportive of an intra-articular pain source and contributed to the decision to proceed with arthroscopic treatment.

The diagnosis of symptomatic cam-type FAI was based on the combination of clinical symptoms, physical examination findings, and radiographic findings rather than on the presence of cam morphology or an isolated radiographic threshold alone. Standardized anteroposterior pelvic and modified Dunn radiographs were routinely obtained. Physical examination included FADIR and FABER testing, with particular emphasis on reproduction of the patient’s symptoms during FADIR testing. Limitation of hip internal rotation, particularly when reduced compared with the contralateral side, was considered an important clinical finding supporting the diagnosis and surgical indication. Cam morphology was assessed radiographically; however, a single alpha-angle cutoff was not used as an isolated diagnostic or surgical criterion, and radiographic findings were interpreted in conjunction with the patient’s symptoms and physical examination findings.

Patient selection for OPHA was deliberately restrictive. Patients with a lateral center-edge angle < 20° were considered to have hip dysplasia and were not considered candidates for this technique. Patients with Tönnis grade ≥ 2 osteoarthritis or clinically relevant joint-space narrowing were also not considered candidates for arthroscopic treatment; therefore, all patients included in the present cohort had Tönnis grade 0 or 1. Similarly, patients with evidence of significant central-compartment chondral pathology on preoperative clinical and imaging assessment were not considered suitable for an only peripheral approach. Concomitant focal pincer morphology was not considered an exclusion criterion when a clinically relevant cam component was present, as focal acetabular overcoverage could be addressed by selective rim trimming and associated labral pathology could be treated during the same procedure. Patients with previous ipsilateral hip surgery were also excluded.

During the study period, 103 patients underwent OPHA. Eight patients underwent revision hip arthroscopy after previous ipsilateral hip arthroscopy performed elsewhere and were excluded because the present study was restricted to primary procedures. Among the remaining 95 patients, 29 were excluded from the final analysis. Twelve patients could not be reached for postoperative follow-up and outcome assessment. Nine patients were excluded because the operative report did not provide sufficient documentation to reliably determine the intraoperative labral status and/or labral treatment. An additional 8 patients were excluded because they were unable to reliably recall their preoperative clinical status and therefore could not provide sufficiently reliable retrospective preoperative PROM data. The final analytic cohort consisted of 66 patients with complete paired preoperative and postoperative outcome data and adequate operative documentation.

The dataset also included operative side, sex, labral treatment category, anchor use, and whether a secondary postoperative procedure was performed. Sex was recorded as documented in the medical record; sex-stratified outcome analyses were not planned because of the sample size.

### 2.3. Surgical Technique

All patients underwent hip arthroscopy without mechanical traction. Patients were positioned supine on a standard operating table, and the operative extremity was draped free to permit unrestricted dynamic hip flexion, extension, abduction, adduction, and rotation throughout the procedure. No traction table or perineal post was used. The surgeon stood on the operative side, and fluoroscopy was used for portal localization and initial access. In the present study, the term ‘traction-free’ refers specifically to the absence of mechanical table traction and perineal-post traction; brief manual traction could be applied when required during labral repair or limited central-compartment visualization.

The procedure was initiated through the peripheral compartment. The anterolateral portal served as the primary viewing portal, while a distal working portal was used for instrumentation. When labral repair or acetabular rim work was required, an additional distal anterolateral accessory portal was created to optimize the trajectory for suture passage and anchor placement. No interportal or T-shaped capsulotomy was performed. Instead, the capsular opening at the working portal was enlarged using a radiofrequency device (Smith & Nephew, Watford, UK) to create an approximately 5-mm working window. When additional working space was required, the capsule was selectively thinned from its intra-articular surface using the radiofrequency device. As no formal capsulotomy was performed, capsular closure was not required and was not performed in any patient.

Femoral osteoplasty was performed for cam morphology under direct arthroscopic visualization. The extent and adequacy of cam resection were determined primarily by dynamic intraoperative assessment rather than by a predefined alpha-angle or fluoroscopic resection target. Because the operative extremity remained freely mobile throughout the procedure, the hip could be repeatedly taken through flexion and rotational movements to assess residual femoroacetabular contact. Osteoplasty was continued until dynamic examination demonstrated impingement-free motion and satisfactory restoration of the femoral head-neck offset. Fluoroscopy was not routinely used to determine the extent of cam resection. A final modified Dunn view was obtained at the completion of the procedure for radiographic documentation.

Concomitant focal pincer morphology was not considered a contraindication to the only peripheral technique. When pincer morphology identified during preoperative assessment was confirmed intraoperatively, selective acetabular rim trimming was performed. The adequacy of rim resection was assessed by direct visualization and dynamic examination rather than by a predefined amount of bony resection.

Labral management was individualized according to tear characteristics and tissue quality. An intact labrum was preserved without intervention. Tears with sufficient tissue quality for fixation were repaired using one to three knotless suture anchors (Smith & Nephew, Watford, UK) according to the extent of the tear, whereas selective debridement was performed for thin, markedly degenerative, unstable, or otherwise nonrepairable labral tissue. When necessary during labral repair, temporary manual traction was applied to facilitate suture passage and limited visualization of the articular surfaces; mechanical traction was not used.

**Figure 1:**
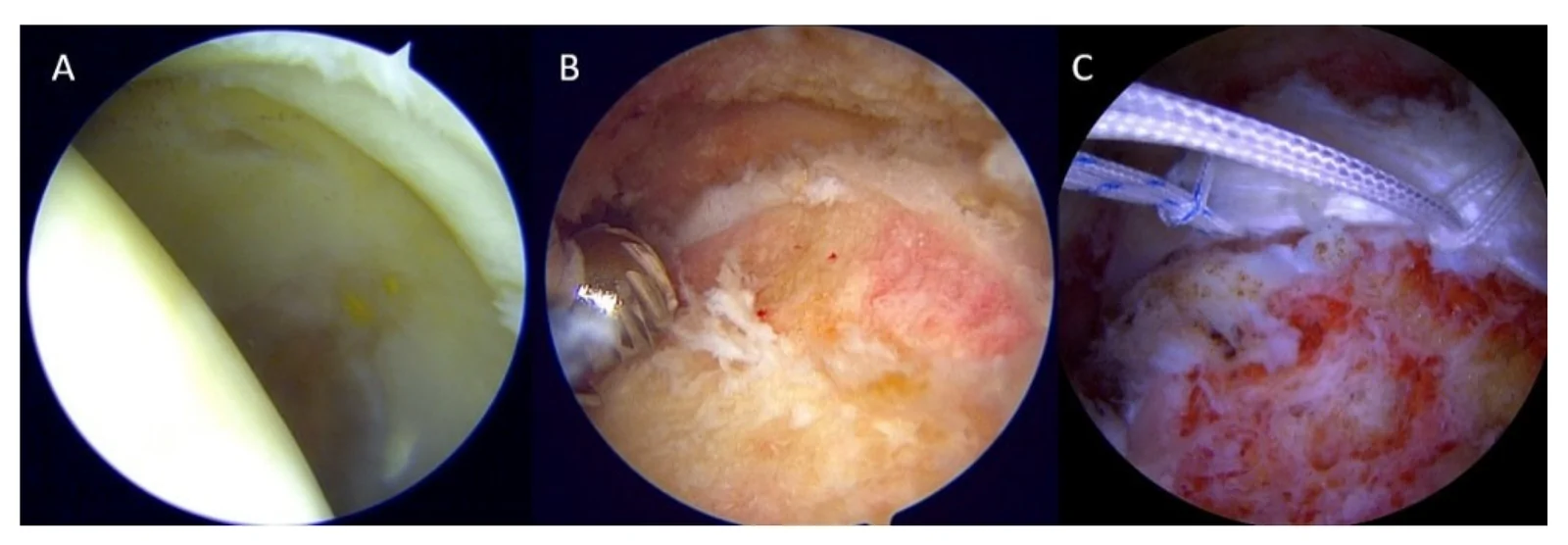
Representative intraoperative arthroscopic images of the traction-free only peripheral hip arthroscopy technique: (**A**) visualization of the central compartment using temporary manual traction; (**B**) final appearance after cam resection; (**C**) arthroscopic appearance after labral repair.

Routine central-compartment access was not performed. Articular cartilage visible during the procedure was assessed and documented; however, central-compartment chondral procedures, including chondroplasty or microfracture, were not performed as part of the OPHA technique. At the completion of the procedure, a final dynamic examination through hip flexion and rotation was performed to confirm impingement-free motion. Representative intraoperative images illustrating temporary manual traction, cam resection, and the final appearance after labral repair are shown in Figure 1.

### 2.4. Postoperative Rehabilitation

Postoperatively, patients were mobilized with two crutches. Weight bearing on the operated extremity was restricted for 2 to 4 weeks, with the duration individualized by the operating surgeon according to the extent of osseous resection performed intraoperatively. In patients who underwent labral repair, hip flexion beyond 90° and excessive rotational movements were restricted during the first 6 weeks. These restrictions were subsequently discontinued progressively according to clinical recovery.

Rehabilitation thereafter focused on progressive restoration of range of motion, muscular strength, and functional activity. Return to running and sport-specific activity was individualized rather than based on a fixed postoperative time point and was allowed progressively after restoration of pain-free range of motion, adequate strength, and functional control. In accordance with contemporary rehabilitation principles following hip arthroscopy for FAI, unrestricted return to sports was generally considered only after completion of progressive functional rehabilitation [12].

### 2.5. Data Collection and Outcome Measures

Demographic and operative variables were extracted from the institutional dataset. Labral status was coded as intact, repaired, or debrided. Clinical outcomes were evaluated using paired preoperative and postoperative values for the VAS pain score, HOS-ADL, HOS-Sport, iHOT-12, and Tegner activity score [13,14,15,16].

Preoperative PROMs were available from the medical records for 55 of the 66 patients. During the early period of the study, standardized PROM collection had not yet been incorporated into routine preoperative clinical assessment; consequently, baseline PROMs were unavailable for 11 patients. As PROM collection subsequently became part of routine clinical practice, contemporaneously recorded preoperative PROMs were available for the majority of the cohort. For the 11 patients without baseline PROMs, the complete PROM questionnaires were administered retrospectively during the long-term outcome assessment, and patients were asked to complete them according to their preoperative pain, function, and activity status. All baseline PROM values analyzed in the study represent individual patient-level questionnaire scores. No transformation, normalization, rounding, imputation, or reconstruction from aggregate summary statistics was performed; the narrow baseline distributions reflect the observed patient-level scores in the study cohort. Patients who were unable to reliably recall their preoperative clinical status were not included in the final cohort. Postoperative outcomes were assessed using the VAS, HOS-ADL, HOS-Sport, iHOT-12, and Tegner activity scales. For patients who did not undergo a secondary intervention, outcomes were obtained at the dedicated long-term follow-up assessment. For patients who subsequently underwent a secondary intervention, the last postoperative PROM assessment obtained before the subsequent procedure or treatment was used in the present analysis. Thus, postoperative outcome scores were not influenced by revision arthroscopy, injection, or conversion to THA. Pain improvement was defined as a reduction in VAS score from preoperative to postoperative assessment. Functional improvement was defined as an increase in HOS-ADL, HOS-Sport, and iHOT-12 scores. For Tegner activity score, positive values indicated increased activity and negative values indicated reduced activity. Subsequent procedures were recorded descriptively according to type, including postoperative injection, revision hip arthroscopy, and conversion to THA, rather than being considered a uniform composite clinical endpoint.

### 2.6. Statistical Analysis

Continuous variables were summarized as mean ± standard deviation and, where appropriate, median with interquartile range. Categorical variables were summarized as frequencies and percentages. Because the outcome variables were ordinal or non-normally distributed, nonparametric tests were used. Preoperative and postoperative paired outcomes within the overall cohort were compared using the Wilcoxon signed-rank test. The same paired analysis was also performed in patients who did not undergo a secondary intervention. Because of the small sample size and heterogeneous nature of subsequent interventions, outcomes in patients who underwent a secondary intervention were summarized descriptively without formal inferential testing. Effect estimates for the principal paired outcome comparisons were reported using Hodges–Lehmann estimates of paired change with 95% confidence intervals.

Labral-treatment subgroup analyses were considered exploratory because of the small and unequal subgroup sizes and differences in underlying pathology. Accordingly, outcomes according to labral status were summarized descriptively without formal post hoc hypothesis testing. For VAS, change was calculated as preoperative minus postoperative score, so a positive value indicated pain improvement. For HOS-ADL, HOS-Sport, iHOT-12, and Tegner activity score, change was calculated as postoperative minus preoperative score. Statistical significance was defined as *p* < 0.05. Analyses were performed using IBM SPSS Statistics, Version 24.0 (IBM Corp., Armonk, NY, USA).

Given the retrospective design of the study, no a priori sample-size calculation was performed. The sample size was determined by the number of consecutive patients who met the predefined eligibility criteria during the study period and had complete paired outcome data.

## 3. Results

### 3.1. Patient Characteristics and Operative Findings

A total of 66 patients were included in the final analysis. The mean age at surgery was 38.9 ± 10.8 years, with a median age of 39.9 years and a range of 18.7 to 62.3 years. The mean postoperative follow-up was approximately 5.7 years (range, 3.7–9.2 years). The cohort included 34 female patients and 32 male patients. The operative side was evenly distributed, with 33 right hips and 33 left hips.

Labral pathology was managed according to intraoperative findings. The labrum was recorded as intact in 32 patients, repaired in 23, and debrided in 11. Anchors were not used in 43 patients, while 13 patients received one anchor and 10 patients received two anchors. Overall, 23 patients underwent anchor-based labral repair.

Eight patients underwent secondary interventions, including four revision arthroscopies, two injections, and two total hip arthroplasties. The two conversions to THA occurred at 6 months and 1 year after the index procedure. Revision hip arthroscopies were performed at 1, 1, 1.5, and 2 years, while postoperative injections were administered at 3 and 4 years. The four patients who underwent revision arthroscopy had baseline Tönnis grades of 0–1, alpha angles ranging from 49° to 75°, and LCEAs ranging from 26.4° to 37.6°; two had concomitant pincer morphology. The two patients who underwent injection both had Tönnis grade 1, with alpha angles of 53° and 62° and LCEAs of 25.9° and 31.9°, respectively, and neither had concomitant pincer morphology. The two patients who subsequently underwent THA were 51 and 62 years old at the index procedure, compared with a mean age of 38.9 years in the overall cohort. Their baseline Tönnis grades were 1 and 0, alpha angles were 72° and 52°, and LCEAs were 35.0° and 31.6°, respectively; both had concomitant pincer morphology. The remaining 58 patients did not undergo a documented secondary intervention (Table 1).

### 3.2. Overall Clinical Improvement After OPHA

In the overall cohort, VAS pain score improved from 7.0 ± 0.6 preoperatively to 3.2 ± 2.5 postoperatively, with a mean improvement of 3.8 ± 2.3 points (*p* < 0.001). HOS-ADL increased from 59.1 ± 1.9 to 79.2 ± 17.5, with a mean improvement of 20.1 ± 16.3 points (*p* < 0.001). HOS-Sport increased from 45.1 ± 2.1 to 67.6 ± 21.3, with a mean improvement of 22.5 ± 20.7 points (*p* < 0.001). iHOT-12 increased from 48.3 ± 1.8 to 72.2 ± 19.4, with a mean improvement of 23.9 ± 18.4 points (*p* < 0.001). Tegner activity score decreased from 5.2 ± 0.7 to 4.3 ± 1.4, corresponding to a mean change of −0.9 ± 1.2 points (*p* < 0.001) (Table 2).

### 3.3. Outcomes in Patients with and Without Secondary Intervention

Among the 58 patients who did not undergo secondary intervention, postoperative outcomes were consistently favorable. VAS improved from 7.0 preoperatively to 2.3 postoperatively, with a mean improvement of 4.7 points (*p* < 0.001). HOS-ADL increased from 59.3 to 82.2, HOS-Sport from 45.3 to 71.2, and iHOT-12 from 48.6 to 75.5, all with statistically significant paired improvements (*p* < 0.001 for each). The Tegner activity score decreased modestly from 5.3 to 4.5 (*p* < 0.001).

Among the eight patients who required a secondary intervention, VAS decreased from 7.6 preoperatively to 6.0 postoperatively, while HOS-ADL remained unchanged at 57.1. HOS-Sport decreased from 43.6 to 41.8, iHOT-12 increased slightly from 46.6 to 48.4, and the Tegner activity score decreased from 4.6 to 2.4. Given the small sample size and heterogeneous nature of the secondary interventions, these findings are presented descriptively without formal inferential testing.

### 3.4. Outcomes Stratified by Labral Treatment

Baseline age, alpha angle, LCEA, and Tönnis grade were broadly similar across the intact, repaired, and debrided labral groups. Pincer morphology was more frequent in the labral repair group, occurring in 0%, 65.2%, and 27.3% of the intact, repaired, and debrided groups, respectively. Given the small and unequal subgroup sizes, these baseline comparisons are presented descriptively.

When outcomes were stratified by labral treatment, the magnitude of change varied across the three groups (Table 3). Among the 32 patients with an intact labrum, VAS improved by 4.6 ± 2.2 points, HOS-ADL by 24.3 ± 14.8 points, HOS-Sport by 27.2 ± 19.6 points, and iHOT-12 by 28.3 ± 17.2 points. The Tegner activity score decreased by 0.7 ± 1.1 points.

Among the 23 patients who underwent labral repair, VAS improved by 3.4 ± 2.2 points, HOS-ADL by 17.7 ± 16.2 points, HOS-Sport by 20.4 ± 20.6 points, and iHOT-12 by 22.3 ± 18.4 points. Tegner activity score decreased by 1.0 ± 1.4 points. Among the 11 patients who underwent labral debridement, VAS improved by 2.5 ± 2.3 points, HOS-ADL by 13.0 ± 18.5 points, HOS-Sport by 13.3 ± 21.8 points, and iHOT-12 by 14.5 ± 19.3 points. Tegner activity score decreased by 1.4 ± 1.2 points.

Patients with an intact labrum showed the greatest numerical improvement in pain, whereas patients undergoing labral debridement showed smaller and less consistent improvement. These findings should therefore be considered exploratory.

## 4. Discussion

The principal finding of this study was that traction-free OPHA was associated with significant improvement in pain and hip-specific functional outcomes in selected patients with cam-type FAI. Improvements were observed in VAS pain score, HOS-ADL, HOS-Sport, and iHOT-12, supporting the clinical relevance of this technique in the studied population. Although the Tegner activity score decreased significantly postoperatively, this finding does not necessarily contradict the improvement observed in HOS-Sport. These instruments assess different aspects of recovery: HOS-Sport reflects hip-related functional ability during sport-related activities, whereas the Tegner scale reflects the level and intensity of activity in which the patient participates. Thus, patients may experience improved hip-specific sports function while returning to activity at a lower overall level or intensity. The significant decrease in Tegner score should therefore be considered a negative finding, indicating that improvement in hip-specific function did not correspond to full recovery of the preoperative activity level. This finding may also have been influenced by retrospective assessment of preoperative activity in a subset of patients and should be interpreted cautiously.

The demographic characteristics of the present cohort should be interpreted in the context of the distinction between cam morphology and symptomatic FAIS. Cam morphology is frequently identified in asymptomatic individuals and therefore should not be considered synonymous with clinically symptomatic disease [17]. In the present study, patients were selected for surgery based on concordant symptoms, clinical examination findings, and imaging abnormalities rather than the presence of cam morphology alone. Although cam morphology has been reported more frequently in male populations [17], sex-related patterns among patients undergoing hip arthroscopy for symptomatic FAIS are more heterogeneous [18]. Accordingly, the nearly equal sex distribution in our cohort (34 female and 32 male patients) likely reflects a selected symptomatic surgical population rather than the epidemiologic distribution of cam morphology in the general population.

Conventional central-compartment-first hip arthroscopy requires joint distraction, and initial access and subsequent instrumentation are performed in close proximity to the articular cartilage and labrum, creating a potential risk of iatrogenic chondrolabral injury [19]. In contrast, OPHA is designed to avoid routine central-compartment entry and to complete cam decompression and selected labral management from the peripheral compartment. This does not diminish the importance of pre-existing cartilage disease; rather, it emphasizes the potential value of minimizing iatrogenic cartilage injury, particularly because cartilage-restoration procedures in the hip remain less predictable than in some other joints [20,21]. Recent comparative evidence has shown that acetabular microfracture is not superior to other cartilage repair techniques and may be associated with equivalent or inferior patient-reported outcomes and equivalent or higher reoperation rates [22]. In addition, patients with small acetabular cartilage defects caused by FAI have not demonstrated clear benefit from microfracture compared with no cartilage treatment [7].

Another potential advantage of OPHA is avoidance of traction-related complications. Because the procedure is performed on a standard table without a perineal post, it avoids perineal compression and prolonged traction as mechanisms of pudendal neurapraxia, lateral femoral cutaneous nerve injury, and other traction-related complications [8,23]. This is clinically relevant because postoperative neuropathy after conventional hip arthroscopy remains an important concern, and traction time and force have been associated with the development and duration of postoperative nerve complications [5]. The traction-free setup also permits dynamic intraoperative evaluation. The surgeon can flex, extend, abduct, adduct, and rotate the hip to assess impingement directly after femoral osteoplasty, thereby confirming correction of the mechanical conflict in real time. However, the present study did not directly assess traction-related neuropraxia or iatrogenic chondral injury and did not include a traction-based control group; therefore, any potential benefit of OPHA regarding these complications remains theoretical and cannot be established from the present data.

The approach to labral management in OPHA is based on the principle that cam morphology should be corrected before definitive labral treatment. In cam-type FAI, the abnormal head-neck relationship can repeatedly irritate the chondrolabral junction [24]. If the labrum is treated without first correcting the underlying bony conflict, the repair or preserved tissue may remain exposed to abnormal mechanical stress [25]. OPHA focuses first on restoring the femoral head-neck offset through direct visualization and dynamic correction. The labrum is then managed according to its condition, including preservation, repair, or selective debridement. Labral preservation and repair may contribute to restoration of the suction seal and hip joint stability [26]. In the present study, improvements in several clinical outcomes were observed across different labral-treatment groups, although the magnitude of improvement varied between groups. However, the greater VAS improvement observed in patients with an intact labrum should be interpreted cautiously. Although age, alpha angle, LCEA, and Tönnis grade were comparable among the labral subgroups, concomitant pincer morphology differed between groups and was more frequent in the labral repair group. The observed difference in pain improvement cannot be attributed to labral status or treatment alone, and these subgroup findings should be considered exploratory.

Patient selection remains particularly important when interpreting outcomes after hip arthroscopy. Older age and pre-existing degenerative changes, including osteoarthritis and cartilage damage, have been associated with less favorable outcomes and an increased risk of conversion to THA after arthroscopic treatment of FAI [27]. In the present cohort, the two patients who subsequently underwent THA were 51 and 62 years old at the index procedure, although neither had advanced radiographic osteoarthritis at baseline. Given the small number of THA conversions, no conclusions regarding predictors of failure can be drawn from the present study.

These findings extend previous experience with OPHA. In a prior study, selected patients with FAI treated with OPHA experienced improved pain and walking ability, improved Postel Merle d’Aubigné scores, reduced alpha angle, and no reported minor or major complications [10]. The present study builds on that experience by including a larger cohort, using hip-specific PROMs, and more clearly defining labral repair and anchor-based stabilization within a traction-free peripheral-compartment strategy. When placed in the context of conventional hip arthroscopy for FAI, the magnitude of improvement observed in the present cohort is broadly consistent with the established literature demonstrating meaningful postoperative improvements in pain and hip-specific function [2]. However, activity recovery appears less complete in the present cohort, as reflected by the significant decrease in Tegner score, whereas systematic reviews of conventional arthroscopic treatment have reported high rates of return to sport [4]. Secondary procedures should likewise be interpreted cautiously: four patients (6.1%) underwent revision arthroscopy and two (3.0%) subsequently underwent THA. These observations provide contextual comparison only, as differences in patient selection, follow-up duration, outcome definitions, and study design preclude direct comparison with published conventional arthroscopy cohorts.

This study has several limitations. First, the retrospective design and absence of a matched control group limit direct comparison between OPHA and conventional traction-based hip arthroscopy. In addition, the inclusion of only patients with complete paired outcome data may have introduced selection bias, as patients with incomplete follow-up or unavailable baseline data were excluded from the final analysis. Second, preoperative PROMs were obtained retrospectively through questionnaire-based recall in 11 of 66 patients (16.7%), whereas contemporaneously recorded preoperative PROMs were available for the remaining 55 patients. The retrospective assessment in this subset represents an important limitation and introduces the potential for recall and response bias, as patients may overestimate or underestimate their previous pain, function, or activity level. Although patients who were unable to reliably recall their preoperative clinical status were excluded, recall bias cannot be eliminated in the 11 patients whose baseline PROMs were reconstructed retrospectively. This should be considered when interpreting the magnitude of the observed preoperative-to-postoperative changes. Third, postoperative PROMs were obtained at variable follow-up intervals, which may have introduced heterogeneity in outcome assessment. This is particularly relevant because postoperative recovery and activity level after hip arthroscopy may vary over time. Fourth, the cohort was heterogeneous with respect to labral pathology and treatment, and the labral subgroups were small and unequal. These subgroup analyses were therefore considered exploratory and may have been confounded by differences in underlying pathology, including concomitant pincer morphology. The small subgroup sizes further limit the precision and interpretability of these descriptive comparisons, particularly in the labral-debridement group. Secondary interventions were also heterogeneous in type and clinical significance. Fifth, although patients with clinically relevant joint-space narrowing or suspected significant central-compartment chondral pathology were excluded during patient selection, quantitative joint-space width was not systematically recorded, and standardized intraoperative grading of central-compartment cartilage was not available for the entire cohort. Although limited central-compartment visualization could be achieved with temporary manual traction when required, particularly during labral repair, manual traction was not routinely applied solely for diagnostic inspection of the articular cartilage. Consequently, central-compartment chondral status could not be systematically graded across all patients. Sixth, sex-stratified outcome analysis was not performed because of sample-size limitations. Finally, secondary interventions were captured from available institutional documentation and may underestimate interventions performed elsewhere. Despite these limitations, the study provides clinically relevant evidence that OPHA may be a feasible traction-free approach for selected patients with cam-type FAI and treatable labral pathology. Finally, secondary interventions were captured from available institutional documentation and may underestimate interventions performed elsewhere.

## 5. Conclusions

Traction-free OPHA was associated with significant improvement in pain and hip-specific functional outcomes in selected patients with cam-type FAI. However, activity levels did not fully recover, as reflected by the significant decrease in Tegner score, and eight patients (12.1%) underwent a subsequent intervention. The technique allows dynamic cam correction and individualized labral management without routine central-compartment distraction or perineal-post traction. Given the retrospective design, retrospective reconstruction of baseline PROMs in a subset of patients, and absence of a matched control group, comparative prospective studies with prospectively collected baseline PROMs are needed to determine whether OPHA provides any advantage over conventional traction-based hip arthroscopy.

## Figures and Tables

**Table 1:** Demographic and operative characteristics of the cohort.

Variable	Overall Cohort, *n* = 66
Age at surgery, years, mean ± SD	38.9 ± 10.8
Age at surgery, years, median [IQR]	39.9 [30.6–47.4]
Age range, years	18.7–62.3
Sex, female/male	34 (51.5%)/32 (48.5%)
Operative side, right/left	33 (50.0%)/33 (50.0%)
Labral status	Intact: 32 (48.5%); repair: 23 (34.8%); debridement: 11 (16.7%)
Anchor use	None: 43 (65.2%); one anchor: 13 (19.7%); two anchors: 10 (15.2%)
Secondary surgery/intervention	8 (12.1%)
Alpha angle	61.0 ± 7.2°
LCEA	34.2 ± 4.2°
Tönnis grade 0	46 (69.7%)
Tönnis grade 1	20 (30.3%)
Concomitant pincer morphology	18 (27.3%)

**Table 2:** Preoperative and postoperative outcomes in the overall cohort.

Outcome	Preoperative Mean ± SD	Preoperative Median (IQR)	Postoperative Mean ± SD	Postoperative Median (IQR)	Mean Change ± SD	Hodges–Lehmann Estimate of Change [95% CI]	Within-Cohort *p* Value
VAS pain score	7.0 ± 0.6	7.0 (7.0–7.0)	3.2 ± 2.5	3.0 (1.0–5.75)	3.8 ± 2.3	4.0 [3.5–4.5]	<0.001
HOS-ADL	59.1 ± 1.9	60.0 (58.0–60.0)	79.2 ± 17.5	84.5 (64.5–96.0)	20.1 ± 16.3	21.0 [17.0–25.0]	<0.001
HOS-Sport	45.1 ± 2.1	45.0 (44.0–46.0)	67.6 ± 21.3	69.5 (51.0–88.75)	22.5 ± 20.7	23.5 [17.5–28.0]	<0.001
iHOT-12	48.3 ± 1.8	49.0 (47.0–49.0)	72.2 ± 19.4	72.5 (57.25–91.75)	23.9 ± 18.4	25.0 [19.5–29.0]	<0.001
Tegner activity score	5.2 ± 0.7	5.0 (5.0–6.0)	4.3 ± 1.4	4.0 (3.0–5.0)	−0.9 ± 1.2	−1.0 [−1.0 to −0.5]	<0.001

Footnote: Values are presented as median [IQR]. The Hodges–Lehmann estimate is reported as the nonparametric effect estimate for paired change, with 95% confidence interval. For VAS, change was calculated as preoperative minus postoperative score, so positive values indicate pain reduction. For HOS-ADL, HOS-Sport, iHOT-12, and Tegner activity score, change was calculated as postoperative minus preoperative score; *p*-values were calculated using the Wilcoxon signed-rank test.

**Table 3:** Preoperative and postoperative outcomes stratified by labral treatment.

Outcome	Labral Group	Preoperative Mean ± SD	Preoperative Median (IQR)	Postoperative Mean ± SD	Postoperative Median (IQR)	Mean Change ± SD
VAS pain score	Intact	7.1 ± 0.5	7.0 (7.0–7.0)	2.5 ± 2.2	2.0 (0.75–3.0)	4.6 ± 2.2
VAS pain score	Repair	7.0 ± 0.6	7.0 (7.0–7.0)	3.6 ± 2.6	4.0 (1.0–6.0)	3.4 ± 2.2
VAS pain score	Debridement	7.0 ± 0.9	7.0 (6.5–7.0)	4.5 ± 2.9	5.0 (2.0–6.5)	2.5 ± 2.3
HOS-ADL	Intact	59.4 ± 1.3	60.0 (59.0–60.0)	83.7 ± 15.4	88.0 (75.25–96.25)	24.3 ± 14.8
HOS-ADL	Repair	59.3 ± 2.0	59.0 (58.0–60.0)	77.0 ± 17.6	77.0 (65.0–94.5)	17.7 ± 16.2
HOS-ADL	Debridement	57.8 ± 2.5	58.0 (56.5–60.0)	70.8 ± 20.6	68.0 (62.5–88.5)	13.0 ± 18.5
HOS-Sport	Intact	45.7 ± 2.4	46.0 (45.0–46.0)	72.9 ± 19.4	72.5 (61.5–90.5)	27.2 ± 19.6
HOS-Sport	Repair	44.9 ± 1.5	45.0 (44.0–46.0)	65.3 ± 21.6	62.0 (50.5–84.5)	20.4 ± 20.6
HOS-Sport	Debridement	44.0 ± 1.9	44.0 (43.0–46.0)	57.3 ± 23.3	55.0 (48.0–72.5)	13.3 ± 21.8
iHOT-12	Intact	48.5 ± 1.6	49.0 (48.0–49.0)	76.8 ± 17.8	78.0 (62.75–93.5)	28.3 ± 17.2
iHOT-12	Repair	48.2 ± 1.7	49.0 (47.0–49.5)	70.5 ± 19.8	69.0 (58.0–90.0)	22.3 ± 18.4
iHOT-12	Debridement	47.9 ± 2.4	47.0 (47.0–49.5)	62.5 ± 20.8	60.0 (54.5–74.0)	14.5 ± 19.3
Tegner activity score	Intact	5.3 ± 0.6	5.0 (5.0–6.0)	4.6 ± 1.3	5.0 (4.0–5.25)	−0.7 ± 1.1
Tegner activity score	Repair	5.2 ± 0.6	5.0 (5.0–5.5)	4.2 ± 1.4	4.0 (3.0–5.5)	−1.0 ± 1.4
Tegner activity score	Debridement	4.9 ± 0.9	5.0 (4.5–5.5)	3.5 ± 1.8	4.0 (2.0–5.0)	−1.4 ± 1.2

## Data Availability

The data presented in this study are available from the corresponding author upon reasonable request. The data are not publicly available because they contain clinical information and are subject to privacy and ethical restrictions.

## Abbreviations

The following abbreviations are used in this manuscript:

**Abbreviation**	**Definition**
FAI	Femoroacetabular impingement
HOS-ADL	Hip Outcome Score activities of daily living subscale
HOS-Sport	Hip Outcome Score sport subscale
iHOT-12	International Hip Outcome Tool-12
OPHA	Only peripheral hip arthroscopy
PROM	Patient-reported outcome measure
VAS	Visual analog scale
